# A rare case of oral multisystem Langerhans cell histiocytosis

**DOI:** 10.4317/jced.53774

**Published:** 2017-06-01

**Authors:** Maria-Teresa Facciolo, Francesco Riva, Patrizia Gallenzi, Romeo Patini, Domenico Gaglioti

**Affiliations:** 1 Oral Surgery Unit of George Eastman Hospital, Umberto I Teaching Hospital, Rome, Italy; 2 Clinical Dentistry Institute of Head and Neck Clinical Area. School of dentistry, Catholic University of Sacred Heart. Largo A. Gemelli, 1 - 00168 Rome, Italy

## Abstract

Langerhans cell histiocytosis (LCH) is a rare disorder characterized by high proliferation of Langerhans dendritic cells. LCH is a solitary or multifocal disease that primarily involves bone tissue and often affects children and young men. A 29 years-old Caucasian man was referred to the Oral Surgery Unit of George Eastman Hospital - Umberto I teaching hospital, with third degree mobility of teeth belonging to second, third and fourth quadrant. Panoramic radiograph showed multiple radiolucent areas with well demarcated borders on the right and left site of the mandible and on the left site of the maxilla. Extractions of compromised teeth and biopsy of the osteolytic tissue were performed. The final diagnosis of multisystem Langerhans cell histiocytosis of the soft and hard tissues of the oral cavity was made. The patient was sent to the Hematology department of Umberto I Teaching Hospital of “Sapienza” – University of Rome for the proper treatment. The present case of rare multisystem LCH involving oral hard and soft tissues shows the strong importance of better investigate, with appropriate additional exams, initial shifty symptoms that could lead to a misdiagnosis.

** Key words:**Differential diagnosis, microscopic diagnosis, Langerhans cell histiocytosis.

## Introduction

Langerhans cell histiocytosis (LCH) is a historically poorly understood hematologic disorder with a wide range of clinical presentations characterized by granulomatous lesions composed of clonal pathologic “histiocytes” (CD207+ cells). LCH may present with a wide spectrum of symptoms, ranging from self-resolving single-organ lesions to disseminated multi-organ disease, which is associated with 10% to 20% mortality and it affects 4 to 8 children per million and 1 to 2 adults per million each year (1,2). Incisional biopsy is fundamental because of the cellular heterogeneity of the lesions and the possible presence of physiologic resident or migrating langerin-positive (CD207+) dendritic cells. The typical architecture of tissues affected by the disease shows a mixture of pathologic dendritic cells and recruited inflammatory cells, including lymphocytes, eosinophils, and macrophages. Pathologic histiocytes have abundant pink cytoplasm, a deep groove in the nucleus that gives them a coffee bean–like appearance, and positive immunohistochemical staining for CD1a, S100 and CD207 (3).

Clinical lesions involve bone, skin, liver, spleen, lymphatic system, bone narrow, lung and oral cavity. Bone and skin are the most commonly involved organs. Bone lesions appear in 75% of patients and skin lesions in 34% (1,2).

Skeletal lesions of LCH often affect skull, long bones, pelvis, ribs, vertebra, facial bones and jaws, particularly the posterior regions of the mandible (4). Oral mucosa involvement is uncommon and it is characterized by gingival hypertrophy and ulcers of the buccal mucosa, hard and soft palate and tongue (5).

The clinical course of the adult onset LCH seems to be unpredictable, but in many cases, it regresses spontaneously or after adequate therapy (6).

This report describes a rare case of multisystem Langerhans cell histiocytosis with oral cavity soft and hard tissues involvement and shows the importance of the integration of radiograph examinations, histological and immunohistochemistry analysis for reaching the correct diagnosis.

## Case Report

A 29 years-old Caucasian man was referred to the Oral Surgery Unit of George Eastman Hospital - Umberto I teaching hospital, with increased mobility of teeth belonging to second, third and fourth quadrant.

The patient had no significant systemic disease, he did not take drugs and his past medical history did not reveal any significant events.

His private dentist had previously extracted the second upper right premolar affected by destructive caries, the second upper left molar, the second and third lower left molars and the second lower right molar affected by severe periodontal disease.

Intraoral examination revealed poor oral hygiene, diffuse gingival inflammation and impairment of the periodontal support with third degree mobility of teeth: 2.6, 2.8, 3.6, 4.6 and 4.7.

**Figure 1:**
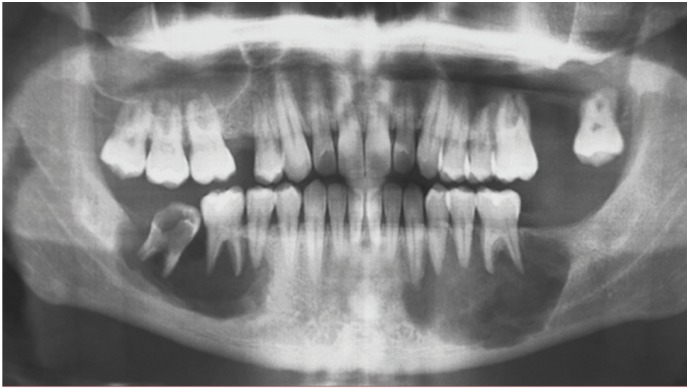
Pre-operative orhopantomography showing well-demarcated radiolucent areas that involve all the dental elements belonging to the third, fourth and sixth sextant.

Panoramic radiograph showed multiple radiolucent areas with well demarcated borders on the right and left mandible and on the left maxilla. The severe alveolar bone resorption of the jaws was located in the region adjacent to teeth from 2.4 to 2.8, from 3.3 to 3.6 and from 4.5 to 4.7 (Fig. 1).

**Figure 2:**
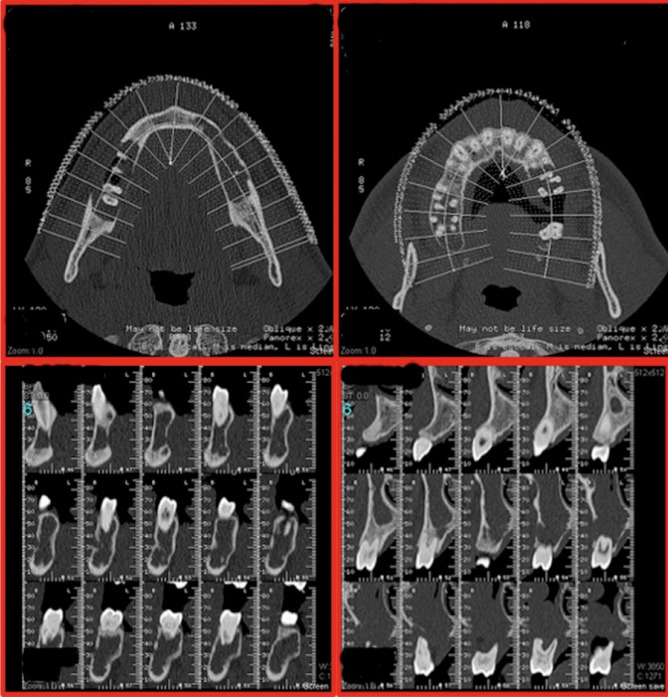
CT scan examination that confirms the presence of osteolytic lesions.

The presence of osteolytic lesions was confirmed by a subsequent 3D examination with CT scan (Fig. 2).

Tooth extractions of 2.8-4.6-4.8 were performed and an incisional biopsy of the osteolytic tissue was sent for histopathological and immunohistochemical examinations to the U.O.C. Pathological Anatomy of S.Spirito Hospital, ASL Roma 1, Rome, Italy.

**Figure 3:**
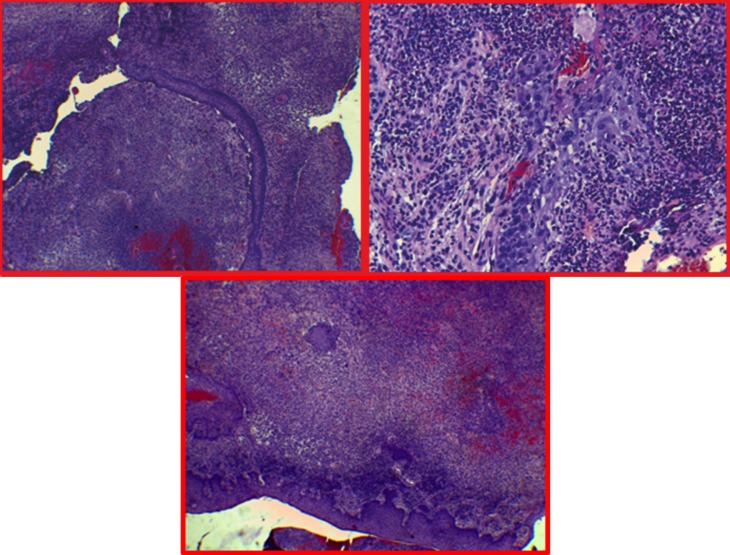
Histologic appearance (Hemat/Eos). Different magnifications. Large cellular infiltrate composed by mononuclear histiocytic cells attacking the epithelium surface with wide ulcerations.

Histological and immunohistochemistry analysis revealed a large cellular infiltrate attacking the epithelium surface with wide ulcerations. The inflammatory infiltrate mainly composed by mononuclear histiocytic cells was positive to S100 protein and to CD1a, CD207, CD68, CD68 (Kp-1) antigen markers (Fig. 3).

According to these findings the final diagnosis of multisystem Langerhans cell histiocytosis of the oral cavity was made.

The patient was sent to the Hematology department of Umberto I Teaching Hospital of “Sapienza” – University of Rome to evaluate the extent of disease and the appropriate treatment.

Complete blood laboratory tests were all within normal limits and infectious disease tests do not show abnormalities.

Chest X-ray confirmed the presence of osteolytic lesions on the right IV,V,VI ribs and on the left III, IV, VII, VIII ribs.

Radiographic examination of both femurs showed oval radiolucent lesion on the upper third of the right femoral diaphysis with perilesional sclerosis also at the level of the right ischiopubic area; osteolytic lesions were detected on the left iliac crest and on the upper third of the left femoral diaphysis, surrounded by a thick margin of reactive bone.

Total body CT scan with technetium-99m methylene diphosphonate (MDP- Tc-99m) revealed regions of increased uptake in the lateral arch of the left VII rib, in the posterior level of the ipsilateral VIII rib, in the posterior arch of the right V rib and in both femoral diaphysis.

A MRI of head highlighted mucositic cysts of the right and left sinus and lack of homogeneity of the cranial bone.

Ultrasonographic examination of the abdomen excluded involvement of “Risk Organs” (Hematopoietic system, spleen, and/or liver) (7).

The presence of multifocal bone lesions and the involvement of the oral mucosa showed the involvement of various organ/system. The final diagnosis of multisystem LCH without involvement of “Risk Organs” was made.

Therapy according to the GIMEMA (Gruppo Italiano Malattie EMatologiche dell’Adulto) LCH 2001 was started. Vinblastine at a dosage of 6 mg/sqm/weekly (10 mg full dose) for a total of six doses combined with Prednisone (PDN) 40 mg/sqm/daily for three weeks then tapered over six weeks was given as induction treatment.

Combined to systemic therapy, an individual oral hygiene program stressing the importance of the correct use of toothbrush, dental floss, inter-dental brushes, and of 0.2% chlorhexidine mouth rinsing to obtain the best possible control of plaque was suggested to the patient.

A causal periodontal treatment with supragingival and subgingival scaling was performed in order to eliminate any source of inflammation.

Subsequently a surgical curettage of the lesions was performed.

During the I phase of treatment the patient underwent supra and subgingival scaling periodically, every two weeks for the first two months and every forty-five days for the following six months.

After six months period of follow-up, a panoramic radiography showed that there was no sign of aggravation of oral lesions.

The patient is currently under treatment and radiographical examinations are scheduled.

## Discussion

A brief literature review conducted by the authors reveals that Langerhans cell histiocytosis is a rare disorder characterized by proliferation of Langerhans dendritic cells.

The pathogenesis of LCH can include oral lesions as well as bone, mucosal and periodontal lesions.

Bone lesions are the most common; they are often localized in an area between the cranium, the maxilla and the mandible. Such lesions can be solitary intra-bony lesion, multiple alveolar lesions, scooped-out alveolar lesions, alveolar lesions with bone sclero-sis or alveolar lesions with bone neoformation. The posterior region of the mandible is considered the most frequently involved region. Mucosal lesions are usually localized in the buccal mucosa and at the back of the vestibule. They present themselves as ulcerated, ovoid or round lesions with erythematous, inflamed borders, painful at the palpation. Such lesions may be associated with lymphadenopathies or with cutaneous lesions. Periodontal lesions like gingival inflammation, ulceration, destruction of the keratinized gingiva, gingival recession, periodontal pockets and bleeding of the oral soft tissues appear as a consequence of the alveolar bone loss. Teeth surrounded by radiolucent defect related to LCH begin to move as “floating teeth” with consequent dental displacement, dental pain and premature loss (8).

The present case of rare multisystem LCH involves oral hard and soft tissues.

The patient shows mucosal erythematous ulcerated lesion, third degree teeth mobility and multifocal lesions involving the mandibular bone and the upper left region of the maxilla. Those lesions were the first manifestation of the LCH disease.

It is important to focus that a third degree teeth mobility in a young patient is a wake up call for the dentists.

A good integration of medical and dental history, clinical examination, radiograph exams, histological and immunohistochemistry analysis is mandatory to reach the correct diagnosis (9).

Histological analysis showed the presence of Langerhans cells and a variable amount of eosinophils, neutrophils, mononuclear and polynuclear histiocytes and lymphocytes. Langerhans cells are oval or rounded in shape, pale, and with a predominantly eosinophilic cytoplasm. Immunohistochemistry analysis revealed that Langerhans cells were positive to S-100 protein, ATP-ase, alpha-D-mannosidase, and antigens CD1a and langerin (4).

The differential diagnosis of multifocal bone lesions of the oral cavity must include odontogenic cysts and tumors, primary bone tumors, osteomyelitis, metastases, multiple myeloma, giant cell granuloma and lymphoma. Only the correct integration of information regarding the medical history, the age, the radiographic exams, the biopsy with histological and immunohistochemistry analysis and blood exams lead the clinicians to the proper diagnosis (9).

The treatment of LCH actually is not clear and it depends on the extent and the severity of the disease.

Single system LCH (SS-LCH) is characterized by the involvement of one organ/system with uni- or multifocal lesions. Such or-gans/systems can be: bone (unifocal - single bone - or multifocal - more than one bone -); skin; lymph node; hypothala-mic-pituitary or central nervous system; lungs (known as primary pulmonary LCH); other (e.g. thyroid or gut). Multisystem LCH (MS-LCH) is characterized by two or more organs/systems involved with or without the involvement of “Risk Organs”.

In SS-LCH the treatment includes surgical curettage, topical injection of steroid, low dose of radiotherapy or observation of spon-taneous regression (6).

In MS-LCH the treatment is characterized by systemic steroidal therapy, immunosuppressant agents, immune modulators or cytostatic drugs (10).

In this case of MS-LCH a systemic therapy with chemotherapy drug vinblastine with oral corticosteroids was prescribed and regular checks to monitor the disease are scheduled.

According to the report of the International Registry of the Histiocyte Society on adult LCH (IRHSA) which studied the clinical characteristics of 274 cases from 13 nations, the probability of survival at 5 years postdiagnosis was 92.3% overall, 100% for patients with single-system disease, 87.8% for isolated pulmonary disease, and 91.7% for multisystem disease. This demonstrates that the number of organs involved is a key factor affecting the prognosis of LCH (11).

Reactivations of LCH in adults occur in about 25-38% of the patients especially in those with multisystem disease (12).

In this case the absence of oral manifestations except teeth mobility showed the importance to better investigate unusual findings such as third degree teeth mobility in a young patient with no history of periodontitis performing radiograph examinations and histological and immunohistochemistry analysis. Only the correct integration of the clinical, radiographic, histological and immunohistochemical data can allow the clinician to reach the final diagnosis and, if necessary, to set up a multidisciplinary treatment plan.

Oral lesions may be the earliest manifestation of the Langerhans cell histiocytosis and in many cases the mouth may be the only site involved. This finding highlights the importance of dentists to make a correct early LCH diagnosis that is of primary importance to improve the patient’s prognosis and quality of life.

## References

[1] Stålemark H, Laurencikas E, Karis J, Gavhed D, Fadeel B, Henter JI (2008). Incidence of Langerhans cell histiocytosis in children: a population-based study. Pediatr Blood Cancer.

[2] Baumgartner I, Von Hochstetter A, Baumert B, Luetolf U, Follath F (1997). Langerhans's cell histiocytosis in adults. Med Pediatr Oncol.

[3] Favara BE, Feller AC, Pauli M, Jaffe ES, Weiss LM, Arico M (1997). Contemporary classification of histiocytic disorders. The WHO Committee On Histiocytic/Reticulum Cell Proliferations. Reclassification Working Group of the Histiocyte Society. Med Pediatr Oncol.

[4] Hicks J, Flaitz CM (2005). Langerhans cell histiocytosis: current insights in a molecular age with emphasis on clinical oral and maxillofacial pathology practice. Oral Surg Oral Med Oral Pathol Oral Radiol Endod.

[5] Milián MA, Bagán JV, Jiménez Y, Pérez A, Scully C, Antoniades D (2001). Langerhans' cell histiocytosis restricted to the oral mucosa. Oral Surg Oral Med Oral Pathol Oral Radiol Endod.

[6] Broadbent V, Pritchard J, Davies EG, Levinsky RJ, Heaf D, Atherton DJ (1984). Spontaneous remission of multi-system histiocytosis X. Lancet.

[7] Zinn DJ, Chakraborty R, Allen CE (2016). Langerhans Cell Histiocytosis: Emerging Insights and Clinical Implications. Oncology (Williston Park).

[8] Dagenais M, Pharoah MJ, Sikorski PA (1992). The radiographic characteristics of histiocytosis X. A study of 29 cases that involve the jaws. Oral Surg Oral Med Oral Pathol.

[9] Eckardt A, Schultze A (2003). Maxillofacial manifestations of Langerhans cell histiocytosis: a clinical and therapeutic analysis of 10 patients. Oral Oncol.

[10] Yashoda Devi BK, Rakesh N, Agarwal M (2012). Langerhans cell histiocytosis with oral manifestations: A rare and unusual case report. J Clin Exp Dent.

[11] Aricò M, Girschikofsky M, Généreau T, Klersy C, McClain K, Grois N (2003). Langerhans cell histiocytosis in adults. Report from the International Registry of the Histiocyte Society. Eur J Cancer.

[12] Girschikofsky M, Arico M, Castillo D, Chu A, Doberauer C, Fichter J (2013). Management of adult patients with Langerhans cell histiocytosis: recommendations from an expert panel on behalf of Euro-Histio-Net. Orphanet J Rare Dis.

